# Maternally sequestered therapeutic polypeptides – a new approach for the management of preeclampsia

**DOI:** 10.3389/fphar.2014.00201

**Published:** 2014-09-05

**Authors:** Gene L. Bidwell, Eric M. George

**Affiliations:** ^1^Department of Neurology, The University of Mississippi Medical CenterJackson, MS, USA; ^2^Department of Biochemistry, The University of Mississippi Medical CenterJackson, MS, USA; ^3^Department of Physiology and Biophysics, The University of Mississippi Medical CenterJackson, MS, USA

**Keywords:** preeclampsia, elastin-like polypeptide, drug delivery, pregnancy, therapeutic peptide

## Abstract

The last several decades have seen intensive research into the molecular mechanisms underlying the symptoms of preeclampsia. While the underlying cause of preeclampsia is believed to be defective placental development and resulting placental ischemia, it is only recently that the links between the ischemic placenta and maternal symptomatic manifestation have been elucidated. Several different pathways have been implicated in the development of the disorder; most notably production of the anti-angiogenic protein sFlt-1, induction of auto-immunity and inflammation, and production of reactive oxygen species. While the molecular mechanisms are becoming clearer, translating that knowledge into effective therapeutics has proven elusive. Here we describe a number of peptide based therapies we have developed to target theses pathways, and which are currently being tested in preclinical models. These therapeutics are based on a synthetic polymeric carrier elastin-like polypeptide (ELP), which can be synthesized in various sequences and sizes to stabilize the therapeutic peptide and avoid crossing the placental interface. This prevents fetal exposure and potential developmental effects. The therapeutics designed will target known pathogenic pathways, and the ELP carrier could prove to be a versatile delivery system for administration of a variety of therapeutics during pregnancy.

## INTRODUCTION

One of the most common complications encountered in obstetrical practice is preeclampsia, occurring in ∼5% of all gestations. Preeclampsia was classically defined as new-onset hypertension and proteinuria, but recent diagnostic criteria released from the American Congress of Obstetricians and Gynecologists (ACOG) has recognized that proteinuria is one of many possible diagnostic criteria (thrombocytopenia, renal insufficiency, impaired liver function, pulmonary edema, or cerebral/visual symptoms) which, when manifested in combination with hypertension, indicate a preeclampsia diagnosis (American College of Obstetricians and Gynecologists and Task Force on Hypertension, 2013). Frustratingly, there is little in the way of pharmacological intervention at the disposal of the physician for the management of the preeclampsia patient, and the only definitive resolution of the disorder is parturition. Current management of these patients is limited to magnesium sulfate for seizure prophylaxis, bed rest, and administration of various anti-hypertensives which typically fail to fully control the progressing hypertension. Unchecked, preeclampsia can potentially develop into eclampsia, which leads to seizures and in some cases, death. Development of new therapeutics for the management of the preeclampsia patient remains an important area of research in obstetrics.

**FIGURE 1:**
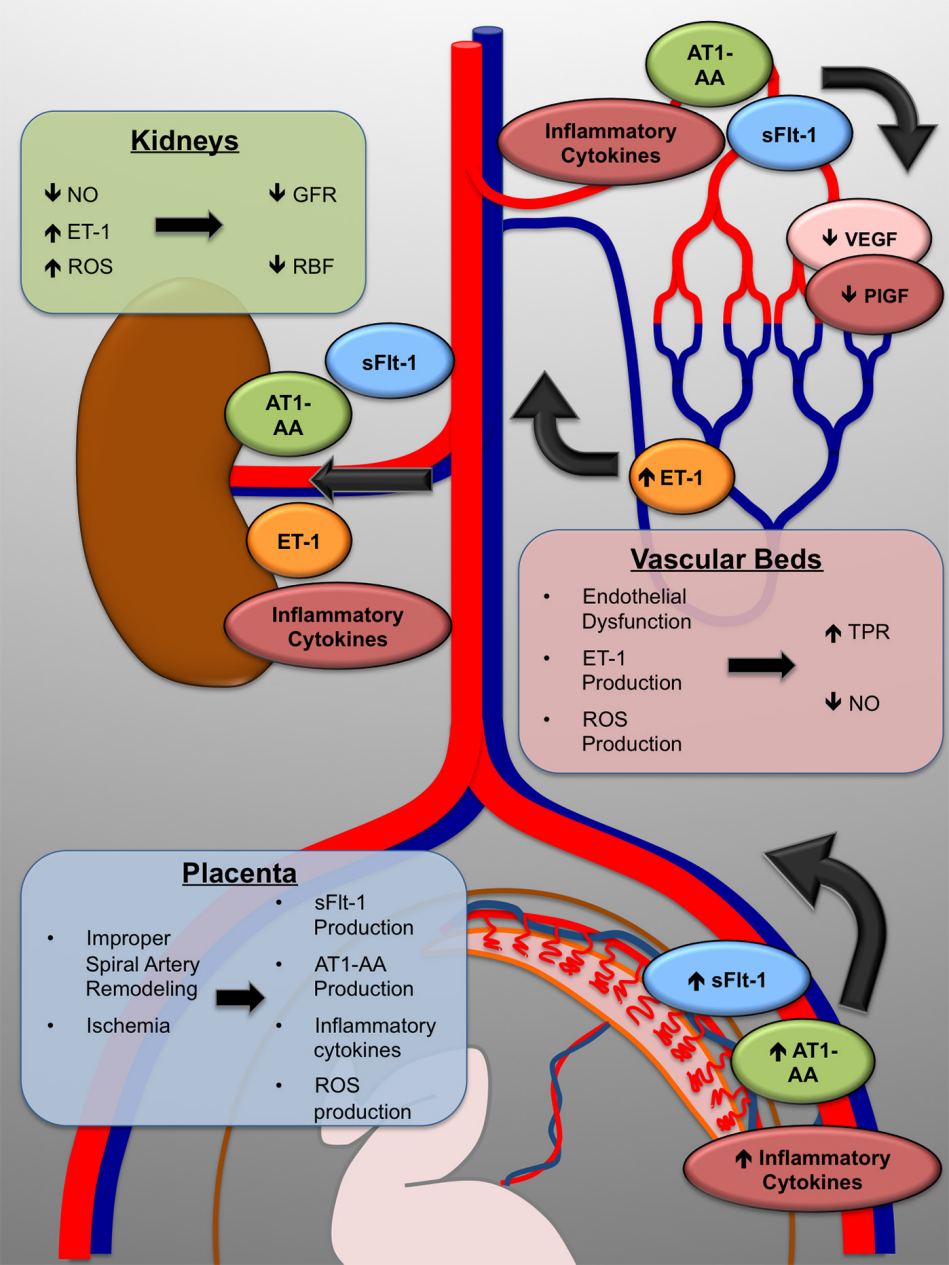
**The maternal symptoms of preeclampsia arise through multiple molecular mechanisms.** Improper placentation leads to placental ischemia. As a direct result, the placenta produces the anti-angiogenic protein sFlt-1, inflammatory cytokines, and increased reactive oxygen species (ROS); as well as increased production of the agonistic AT-1 receptor autoantibody (AT1-AA). The maternal vasculature, including that in the kidneys, is exposed to decreased VEGF signaling and inflammatory mechanisms which cause endothelial dysfunction, marked by overproduction of the vasoconstrictor endothelin-1 (ET-1). In the kidneys, total peripheral resistance (TPR) increases, renal blood flow (RBF) and glomerular filtration rate (GFR) decrease, and maternal hypertension is the end result.

While a great deal of research has begun to elucidate the molecular and physiological mechanisms which are responsible for the maternal symptoms, the initiating causes remain unclear. What has become generally accepted is that the disorder is closely linked to defects at the maternal/fetal interface, particularly in the remodeling of the maternal spiral arteries which supply the blood flow to the placenta (Khong and Brosens, 2011). During gestation, the developing fetus requires copious amounts of blood flow to the placenta to allow for adequate exchange of nutrients and wastes between the maternal and fetal circulations. To ensure adequate delivery of blood, the maternal spiral arteries of the uterus undergo a dramatic remodeling. Fetally derived cytotrophoblasts invade the maternal vessels, displace the endothelium, and convert the normally small diameter, low capacitance vessels into dilated high capacitance vessels. Clues that the placenta was central to the etiology of preeclampsia came from case reports showing that delivery of the fetus alone was insufficient to remit the disease symptoms, and that delivery of the placenta was crucial for resolution (Shembrey and Noble, 1995). Early histological examination of placentas from preeclampsia patients suggested that the remodeling of these arteries in preeclampsia patients was deficient, with only very shallow trophoblast invasion and arterial remodeling. This led to the idea that in preeclampsia, the placenta-which even in normal pregnancy is relatively hypoxic-receives inadequate blood flow and in consequence experiences chronic hypoxia and ischemia. Indeed, a host of studies over the last 15 years have strongly implicated placental ischemia as a central factor in the manifestation of preeclampsia. Research into the molecular links between chronic placental ischemia and the symptomatic phase of the disorder continue, but several pathways have been intensively investigated and validated. This includes production of the anti-angiogenic protein soluble fms-like tyrosine kinase-1 (sFlt-1), production of inflammatory cytokines such as TNFα, and increased production of oxidative stress in the placenta and maternal vasculature (**Figure 1**).

## PATHWAYS DRIVING PREECLAMPSIA

### ANGIOGENIC IMBALANCE

One of the pivotal breakthroughs in the understanding of preeclampsia was the recognition of increased circulating levels of the vascular endothelial growth factor (VEGF) antagonist sFlt-1 in the circulation of preeclampsia patients (Maynard et al., 2003). sFlt-1 is a soluble, alternatively transcribed isoform of the VEGF receptor Flt-1 and consists only of the receptor’s recognition domain. This soluble protein is then secreted extracellularly, where it competes for VEGF binding, thus making VEGF unavailable to bind to its full length, active receptors (Wu et al., 2010). While the mechanisms which regulate sFlt-1 splicing are still under investigation, a number of preclinical studies have shown that production of sFlt-1 from placental tissue is increased by either *in vivo* chronic ischemia, or acutely by hypoxia *ex vivo* (Ahmad and Ahmed, 2004; Nagamatsu et al., 2004; Nevo et al., 2006; George et al., 2010).

A variety of studies have supported a link between loss of VEGF activity and hypertension. Patients receiving the anti-VEGF antibody therapy bevacizumab experience hypertension and proteinuria – side effects which are remarkably similar to preeclampsia patients (Zhu et al., 2007). Likewise, inhibition of the VEGF receptors by small molecule tyrosine kinase inhibitors increases blood pressure, at least partially mediated by increased endothelin-1 expression – a known final effector of hypertension in preeclampsia patients (Kappers et al., 2010, 2011, 2012; George and Granger, 2011). Finally, a plethora of studies have demonstrated that increasing circulating sFlt-1 levels through direct administration or viral overexpression induces a hypertensive, preeclampsia-like phenotype in animal models (Maynard et al., 2003; Li et al., 2007; Bridges et al., 2009; Suzuki et al., 2009; Gilbert et al., 2010; Murphy et al., 2010). sFlt-1 has therefore become a major target of interest, and a recent study has shown beneficial effects of sFlt-1 removal by apheresis in a small cohort of preeclampsia patients (Thadhani et al., 2011). Therapeutics targeting sFlt-1 to restore angiogenic balance are a promising avenue for drug development.

### THE MATERNAL INFLAMMATORY RESPONSE

Another well-characterized mechanism which has been extensively studied is the production of inflammatory cytokines in response to placental ischemia/hypoxia. Recent research has revealed that inflammatory processes play an important role in the etiology and progression of preeclampsia (Borzychowski et al., 2006; Ahn et al., 2011). The placenta is home to a variety of hematopoietic cells, including T cells, natural killer (NK) cells, and macrophages, and all have roles in production of cytokines including TNF-α and pro-inflammatory interleukins that exacerbate the immune response in preeclampsia (Azizieh et al., 2005). This highly inflammatory environment is a double-edged sword. High INF-γ and TNF-α levels inhibit trophoblast migration and are directly toxic to trophoblasts (Yui et al., 1994; Todt et al., 1996; Rasmussen et al., 1999), so they may contribute to the initial improper remodeling that leads to preeclampsia. Also, TNF-α and other inflammatory factors induce systemic endothelial dysfunction, including increased endothelin-1 release, induction of oxidative stress, and enhanced sensitivity to angiotensin II (AngII), which combine to exacerbate the maternal hypertension (Gilbert et al., 2008).

Of all the inflammatory cytokines examined, perhaps none have been as consistently described and characterized as TNF-α. Elevated TNF-α levels have been described in both the maternal circulation and amniotic fluid of preeclampsia patients (Kupferminc et al., 1994; Vince et al., 1995) as well as in the placenta and circulation of rodents undergoing placental ischemia (LaMarca et al., 2008). In rats, blockade of TNF-α signaling by etanercept partially attenuates the hypertension associated with placental ischemia, and infusion of TNF-α to levels seen in rodents with placental ischemia leads to a hypertensive phenotype associated with increased vascular production of endothelin-1 (LaMarca et al., 2005, 2008). Furthermore, one of the most recently elucidated pathways in preeclampsia is the production of agonistic auto-antibodies to the angiotensin type 1 receptor (AT1-AA) which are found in a large percentage of preeclampsia patients (Xia et al., 2003; Herse et al., 2009). Interestingly, the AT1-AA has been shown to induce the production of TNF-α in pregnant mice, suggesting that it might be one of the upstream regulators of TNF-α production in preeclampsia patients (Irani et al., 2010). These data and others suggest that TNF-α is an important regulator of the symptoms associated with preeclampsia and placental ischemia. Targeting of the inflammatory cascade set off by increased TNF-α levels could be an important target in the development of preeclampsia therapeutics.

### OXIDATIVE STRESS

One other known player in the response to placental ischemia is the production of reactive oxygen species (ROS). The ischemic environment of the preeclamptic placenta has been shown to induce ROS production, (Staff et al., 1999a,b) either as a direct consequence of hypoxia or as a secondary response to the local inflammatory environment. Additionally, ROS production may also be induced in the systemic vasculature due to the highly inflammatory environment (Roggensack et al., 1999). Superoxide is the major ROS produced in the preeclamptic placenta, and its production might be a consequence of the action of the mitochondrial electron transport chain enzymes, xanthine oxidase, or NADPH oxidase (Nox) operating under low oxygen conditions (Myatt, 2010). Superoxide can act locally as a damaging oxidant, it can dismute to hydrogen peroxide, or it can react with nitric oxide to produce peroxynitrite (Myatt, 2010).

The increase in ROS in both the placenta and in the systemic vasculature might play a role in the development of PE symptoms. Within the placenta, superoxide and peroxynitrite act locally at the site of production in the vascular endothelium and surrounding stroma to induce damaging protein oxidation, lipid peroxidation, or protein nitration (Myatt, 2010). Systemically, ROS (produced by neutrophils (Lee et al., 2003a,b) or directly in vascular endothelial cells) can further exacerbate endothelial dysfunction, leading to endothelin-1 production and reduced NO bioavailability, which ultimately lead to hypertension via increased total peripheral resistance (TPR; George and Granger, 2011). In addition to the direct induction of ROS production in the placenta and the systemic vasculature, women with PE also have decreased superoxide dismutase (SOD), glutathione, and glutathione peroxidase levels and impaired SOD activity (Wang and Walsh, 2001). Therefore, they may have a reduced antioxidant capacity and thus a heightened response to the ROS production relative to a normal pregnant mother (Myatt and Cui, 2004). Supporting ROS as a target for intervention, rats with hypertension resulting from placental ischemia or sFlt-1 excess have significantly decreased blood pressure when administered anti-oxidant compounds (Sedeek et al., 2008; Tam Tam et al., 2011). These data suggest that ROS is a major contributor to the symptomatic manifestation of preeclampsia and that target modulation of ROS could be a potential therapeutic approach for the preeclampsia patient.

## THERAPEUTIC STRATEGIES TARGETING THE MECHANISMS DRIVING HYPERTENSION IN PREECLAMPSIA

Above, we have outlined the evidence supporting ischemia-induced placental production of sFlt-1, activation of the innate immune system, and induction of ROS production in the symptomatic manifestation of preeclampsia. Elucidation of these pathways that clearly drive the symptomatic phase of the disease lead us to hypothesize that interventions targeted to these pathways could be effective therapies for preeclampsia. Specifically, we hypothesize that supplementation with exogenous VEGF or placental growth factor (PlGF) to restore the depressed levels and sequester the overabundant sFlt-1 will have a positive effect on maternal hypertension, and as a result, fetal health both at birth and later in life. Similarly, we hypothesize that inhibition of the inflammatory pathway by inhibition of its master mediator NF-κB will serve to improve both maternal symptoms and fetal outcomes. Finally, we suggest that selective inhibition of enzymes responsible for ROS production could be beneficial for PE therapy. However, in order to achieve these outcomes, novel sFlt-1, NF-κB, and ROS inhibiting agents must be stabilized from degradation in the maternal circulation and ideally be prevented from crossing the placental interface and entering the fetal circulation, where they could be harmful to the developing fetus.

### THE ELP DRUG DELIVERY SYSTEM

**FIGURE 2:**
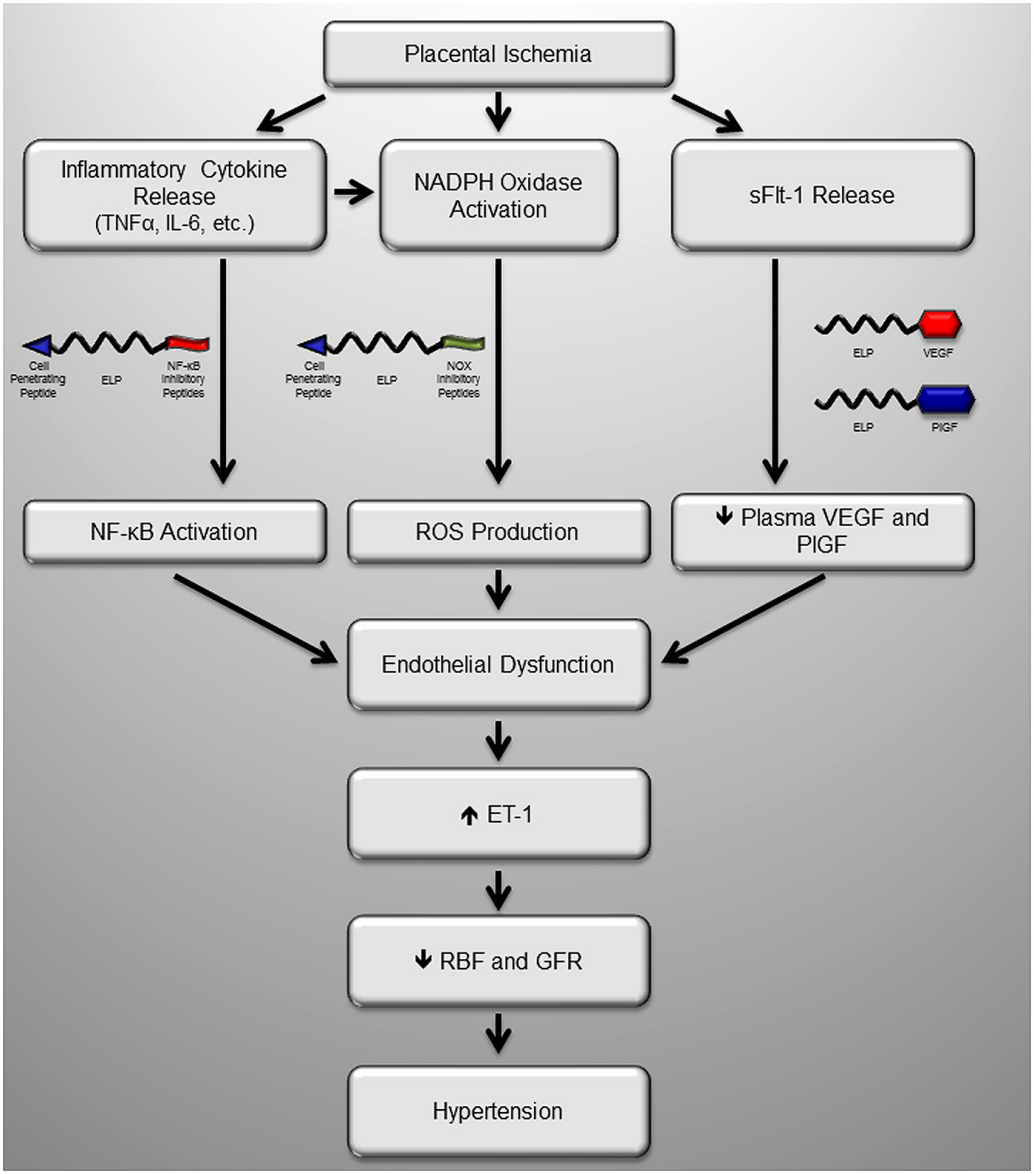
**Potential sites of intervention with maternally restricted therapeutic peptides during preeclampsia.** We have recently produced several novel maternally restricted peptide therapeutics to target known pathologic molecules produced in response to placental ischemia in preeclampsia. Much of the activity of inflammatory cytokines is through the master regulator NF-κB, which could be blocked by the inhibitory ELP-p50 peptide which blocks nuclear translocation of NF-κB, a transcription factor. The major source of oxidative stress in the ischemic placenta is the NADPH oxidase (Nox) enzyme, which could be inhibited by the Nox inhibitory peptide, which blocks assembly of the complete complex. Finally, one of the most extensively characterized mechanisms is production of the VEGF inhibitor sFlt-1, which act as a decoy receptor. Introduction of ELP-stabilized VEGF or PlGF fusions would restore VEGF signaling by their direct activity, and/or by sequestering the excess sFlt-1 in the maternal circulation. Ultimately all of these proposed peptides help to restore endothelial function and renal function to block the maternal hypertension.

Our group has recently been developing a carrier protein called elastin-like polypeptide (ELP) for use as a drug delivery vector during pregnancy. ELP is a genetically engineered polypeptide consisting of repeated units of a five amino-acid motif (VPGxG, where x is any amino acid except P; Urry et al., 1991a). ELP has a unique property of reversibly forming aggregates in response to heat. Above a characteristic transition temperature, the polypeptide will form aggregates, and when the solution is lowered below the transition temperature, the aggregates re-dissolve (Urry et al., 1991a). There are several advantages of using ELP polypeptides for drug delivery. First, ELPs are genetically encoded rather than chemically synthesized. The coding sequence for ELP is built into a plasmid-based recombinant expression system. This means the researcher has absolute control over the ELP sequence and molecular weight (MW). Changes to the ELP sequence or modification of the number of ELP repeats are achieved by simple molecular biology techniques. This is important because the size of a polymer carrier influences the plasma pharmacokinetics and tissue distribution (Dreher et al., 2006), and because the size and sequence of ELP influences the transition temperature of its heat-induced aggregation (Urry et al., 1991a). Also, addition of targeting peptides and therapeutic proteins is easily achieved by modifying the DNA coding sequence. Second, ELP and ELP-fusion proteins can be expressed in *E. coli*, and large quantities of the molecules can be purified by simply taking advantage of the thermal responsiveness. Purification of ELP-fusion proteins is achieved by heating a bacterial lysate containing the recombinantly expressed ELP to a temperature above the polypeptides’ transition temperature. This induces ELP aggregation, and it is collected by centrifugation (Meyer and Chilkoti, 1999; Bidwell and Raucher, 2005). Repeated centrifugation above and below the transition temperature leads to large quantities of very pure protein (Meyer and Chilkoti, 1999; Bidwell and Raucher, 2005). The third advantage for using ELP for drug delivery is that it is a large, biologically inert macromolecule. Therefore, ELP fusion can stabilize protein, peptide, or small molecule cargo in systemic circulation (Bidwell et al., 2012, 2013), and targeting agents can be used to direct the ELP-fused therapeutics’ biodistribution (Bidwell et al., 2009). Previous work has used ELPs extensively for drug delivery in cancer models. These studies have carefully defined the polypeptide’s pharmacokinetics and biodistribution (Liu et al., 2006; Bidwell et al., 2012, 2013; Moktan et al., 2012), confirming ELP as a long circulating, inert, biodegradable, and non-immunogenic (Urry et al., 1991b) drug carrier. In addition, our previous cancer work also serves as proof of principle for the ability to ELP to efficaciously deliver active therapeutic peptides (Bidwell et al., 2012, 2013). Recently, we have found that ELP has a long half-life after intravenous injection in a rat pregnancy model, but importantly, ELP does not cross the placenta (George et al., 2014). Our current goals are to leverage this powerful delivery system to (1) stabilize therapeutic proteins, peptides, or small molecules targeted to preeclampsia mechanisms in the maternal circulation and (2) prevent the penetration of these therapeutic agents into the developing fetus. We are currently developing ELP-fused therapeutics to target all three pathways described above using ELP-fused to VEGF or PlGF, ELP-fused peptide inhibitors of NF-κB, and peptide inhibitors of NOX (**Figure 2**).

The ELP delivery system can be utilized in either an untargeted manner, which is useful when attached to agents designed to circulate, or it can be targeted to enter cells or even to specific intracellular compartments using cell penetrating peptides (CPPs; Massodi et al., 2005; Bidwell et al., 2009). CPPs do not necessarily provide tissue specificity, but they do enhance deposition of ELP in many of the major organs, most notably the kidney (Bidwell et al., 2013). For delivery of VEGF or PlGF, we are utilizing a version of the polypeptide that is untargeted. This will allow the ELP-VEGF or ELP-PlGF to freely circulate, where it can bind and sequester the excess s-Flt-1. For delivery of the NF-κB inhibitory peptides or the NOX inhibitory peptides (which have intracellular targets), we are utilizing ELP vectors fused to CPPs to mediate cellular uptake of the polypeptide and direct them to the cytoplasm. We have previously shown that multiple CPPs are effective for intracellular delivery of ELP both *in vitro* (Massodi et al., 2005) and *in vivo* (Bidwell et al., 2012, 2013; Moktan et al., 2012).

In a recent study, we demonstrated that ELP and a CPP-tagged ELP are excluded from the fetus after systemic administration to the mother in a rat pregnancy model (George et al., 2014). Furthermore, this fetal exclusion held even after 5 days of continuous infusion of the polypeptides. Within the placenta, both ELP and CPP-tagged ELP were detectable in the cytoplasm of trophoblast cells, but were absent from the fetal portion of chorionic villi. We believe that the size of the ELPs prevents them from crossing the tight junctions between trophoblast cells in the chorionic villi.

### NOVEL THERAPIES TARGETING THE sFlt-1/VEGF PATHWAY

As described above, one of the hallmarks of preeclampsia is the elevated maternal plasma levels of the VEGF antagonist sFlt-1. We hypothesize that VEGF or PlGF supplementation therapy could be a viable mechanism to sequester the excess sFlt-1 and thereby prevent or reverse the onset of hypertension. While we believe that VEGF supplementation therapy will be beneficial for treatment of preeclampsia, the therapeutic strategy is not as straightforward as simply infusing VEGF. VEGF infusions have been tested in several disease models, and this approach has been hindered by many problems. First, free exogenous VEGF is very short-lived, with a plasma half-life in humans of about 34 min (as determined following a four hour intravenous infusion of recombinant human VEGF_165_; Eppler et al., 2002). Exogenous VEGF therapy has shown potential in several disease models, including myocardial infarction (Banai et al., 1994; Pearlman et al., 1995), but due to the short half-life and poor stability of the protein, constant infusion via a pump-driven catheter placed directly at the diseased site was required. This type of treatment strategy is not a viable translational approach for preeclampsia therapy, where patients will need to be treated for several months.

The second limitation of free VEGF supplementation for preeclampsia therapy involves its potential for damage to the developing fetus. Several reports have demonstrated the severe potential consequences of overloading the fetus with VEGF. Overexpression of VEGF-A by two to threefold using a genetic strategy in mouse embryos resulted in embryonic lethality at day E12.5 (Miquerol et al., 2000). Death was due to cardiac failure resulting from malformation of the myocardium, improper ventricular septation, and abnormalities in the heart’s outflow track. A separate study in which quail embryos were directly injected with exogenous VEGF showed similar results. The VEGF treated embryos had neovascularization in normally avascular regions and excessive, improper fusion of vessels (Drake and Little, 1995). Like the mouse study, these VEGF treated embryos also had malformation of the hearts, including fusion of inflow and aortic outflow channels. These studies address the dire consequences of increasing VEGF levels directly in the developing fetus, but it has also been shown that administration of free VEGF to pregnant mice causes developmental problems in the embryos. Daily systemic injection of recombinant human VEGF from gestational day 9–day 17 resulted in an 18-fold increase in the fetal reabsorption rate and a significant decrease in fetal weight among the surviving fetuses (He et al., 1999). Given the limitations of short half-life and the potential for teratogenic effects of free VEGF, we have fused VEGF (or similarly PlGF) to the ELP carrier to both extend its plasma half-life and prevent its delivery across the placenta. The goal of this strategy is to supplement circulating VEGF or PlGF levels in order to achieve levels present in normal pregnancy. We have recently characterized the ELP-VEGF polypeptide and found that ELP-VEGF is equally potent at stimulating vascular endothelial cell proliferation, migration through a collagen matrix, and tube formation when compared to unbound VEGF (unpublished data). Given the favorable biodistribution profile of ELP-fused VEGF or PlGF and a careful dosing strategy to achieve proper VEGF or PlGF replacement, we believe that this approach represents a promising new method for preeclampsia therapy.

### NOVEL THERAPIES TARGETING THE INFLAMMATORY PATHWAY

Many pro-inflammatory cytokines such as TNF-α, IL-1, and toll like receptors (TLRs, whose signaling has also been implicated in PE (Keelan and Mitchell, 2007; Tinsley et al., 2009; Chatterjee et al., 2012) exert their effects via receptor-mediated signaling pathways that are centrally routed through NF-κB. In fact, NF-κB is a master regulator of inflammation (Makarov, 2001). For this reason combined with the multitude of inflammatory factors at play, we chose to target NF-κB as a means to inhibit the inflammation associated with preeclampsia. Several previous studies have highlighted the importance of NF-κB activation in preeclampsia. Hypoxia and reoxygenation of villous explants leads to activation of the NF-κB pathway (Cindrova-Davies et al., 2007), and NF-κB activation plays an important role in the systemic endothelial dysfunction present in preeclampsia (Jiang et al., 2010). Furthermore, analysis of microarray data of placental tissue from preeclamptic women versus at-risk but non-preeclamptic controls reveals NF-κB as a major pathway that is upregulated in preeclampsia (Centlow et al., 2011). Within the placenta, NF-κB levels in preeclampsia are associated with increased trophoblast apoptosis (Aban et al., 2004), and systemic NF-κB activation is also present in the vasculature of preeclamptic mothers (Shah and Walsh, 2007). Systemic vascular NF-κB activation is associated with neutrophil infiltration, and these neutrophils release toxic substances such as TNF-α, ROS, and thromboxane, which promote vasoconstriction and vascular dysfunction (Shah and Walsh, 2007).

NF-κB activation is a complex pathway involving trimerization of NEMO, the regulatory domain of inhibitor of κ-B kinase (IKK), phosphorylation and deactivation of the NF-κB inhibitor I-κB by IKK, NF-κB phosphorylation on the p65 subunit, and active import of NF-κB into the nucleus (reviewed in Bidwell and Raucher, 2009). Extensive interest in developing NF-κB inhibitors exists in the cancer field, and several previous studies have described peptide inhibitors of all the activation steps mentioned above (Bidwell and Raucher, 2009). We have developed an ELP-fused peptide inhibitor of activated NF-κB. NF-κB activation upon extracellular signaling is mediated by phosphorylation and release of the natural inhibitor I-κB from the NF-κB p50/p65 heterodimer. I-κB release exposes a nuclear localization sequence (NLS) on the p50 subunit of NF-κB, and once exposed, this NLS mediates nuclear import of NF-κB. Once inside the nucleus, NF-κB binds to response elements on its target genes and regulates gene expression. A synthetic cell permeable peptide containing the p50 NLS is capable of blocking the nuclear import of NF-κB upon stimulation in a variety of cell lines (Lin et al., 1995). We have fused a copy of the p50 NLS to the CPP-ELP carrier and validated its activity *in vitro*. We are currently focusing on determining the pharmacokinetics and biodistribution of this NF-κB inhibitory polypeptide, and assessing its efficacy in our rat models of PE. Initial biodistribution data have shown that the CPP-ELP-delivered p50 NLS peptide accumulates highly in the kidneys and the placenta. This biodistribution is advantageous because these two organs are the most critical for modulating the drivers of hypertension and proteinuria in preeclampsia. We hypothesize that the polypeptide will function to inhibit NF-κB in these tissues (either in the tissue stromal cells directly or in invading lymphocytes) and reduce the local inflammatory environment.

### NOVEL THERAPIES TARGETING REACTIVE OXYGEN SPECIES PRODUCTION

Of the possible sources listed above, the Nox family is thought to be the major contributor to ROS production in the placenta. Nox1 expression has been found in syncytiotrophoblasts and vascular endothelial cells in the placenta (Matsubara and Sato, 2001; Cui et al., 2006), and its levels are enhanced in tissue from PE patients (Cui et al., 2006). Elevated total Nox activity has also been seen in placentas from women with early onset PE, though no difference was seen when comparing all PE patients to normal pregnant controls (Raijmakers et al., 2004). It has also been demonstrated that ROS are produced via Nox2 in vascular smooth muscle cells and trophoblasts in response to the AT1-AA, and ROS production induced NF-κB activation (Dechend et al., 2003). The AT1-AA also induced Nox2 subunit production and ROS production in pregnant rats (Parrish et al., 2011). Furthermore, treatment of vascular endothelial cells with sera from preeclampsia patients induced production of the Nox2, an effect attenuated by an Ang II type 1 receptor antagonist (Matsubara et al., 2010).

We hypothesize that Nox inhibition could be a viable therapeutic strategy for PE, either alone or in combination with targeting other pathways. Patrick Pagano’s lab has described a number of peptide inhibitors of the Nox family (reviewed in (Cifuentes-Pagano et al., 2012). The most specific and widely used agent, called Nox2 docking sequence (Nox2ds) or gp91ds, is an nine amino acid peptide that inhibits the interaction between Nox2 and its partner in the complex p47*^phox^* (Rey et al., 2001). The Nox2ds peptide, fused directly to the Tat CPP, has been shown to inhibit superoxide production in a variety of cell and tissue types in response to multiple stimuli. The peptide has been used extensively in many disease models including AngII-induced hypertension, renovascular hypertension, and arterial balloon injury (reviewed in Cifuentes-Pagano et al., 2012). For application in PE, we fused the Nox2ds peptide to the CPP-ELP carrier (Bidwell and Raucher, 2009). The CPP-ELP carrier will increase the plasma half-life relative to the free peptide, and the use of the CPP will mediate uptake of the polypeptide into target cells in the placenta or systemic vasculature. We are currently testing the ability of this polypeptide to inhibit placental and/or vascular ROS production in our rat models of PE.

## CONCLUSION

The explosion of research into the etiology of preeclampsia has provided a number of intriguing targets for therapeutic development. The ideal scenario would be to understand the pathways that lead to improper spiral artery remodeling and intervene very early in pregnancy to prevent the improper remodeling. However, in the absence of this knowledge and in the absence of a concrete biomarker to predict which patients will develop preeclampsia, this type of intervention is currently unrealistic. In contrast, we now know of many of the molecular pathways that lead to the precipitation of the symptomatic phase of preeclampsia. Our aim is to intervene during this symptomatic phase and modulate these pathways of interest with the goal of prolonging gestation and thereby improving fetal outcomes. While small molecule therapeutics are one potential method to affect these pathways, great care must be exercised in their development, as unwanted and potentially harmful effects to the fetus are possible. Peptide-based therapeutics, though an active area of research in cancer therapy, also have potential as therapeutics in cardiovascular disorders. However, free peptides suffer from very rapid plasma clearance and susceptibility to degradation *in vivo*. Our goal is to develop an appropriate polymeric carrier that can be fused to either small molecule drugs or to therapeutic peptides/proteins. By fusing these agents to the carrier, they can be stabilized in the maternal circulation and prevented from entering the fetal circulation. We feel that the proposed therapeutic agents, or agents like them that intervene in pathways of known importance in preeclampsia (angiogenic factors, maternal inflammatory/autoimmune response, and oxidative stress), have great promise as maternally sequestered therapeutics. Future studies will explore the therapeutic potential of these agents in the management of preeclampsia.

## Conflict of Interest Statement

The authors hold provisional patents on the described therapeutics.
